# Preserved Spontaneous Ventilation for Tracheostomy in a Severely Agitated Patient With Bilateral Vocal Cord Paralysis: A Case Report

**DOI:** 10.7759/cureus.117223

**Published:** 2026-09-30

**Authors:** Tatiana Salloum, Rohan Dureja, Waleed Cassis, Gisele Wakim

**Affiliations:** 1 Department of Anesthesiology, Perioperative Medicine, and Pain Management, University of Miami Miller School of Medicine, Miami, USA

**Keywords:** bilateral vocal cord paralysis, difficult airway, emergency tracheostomy, ent procedures, otolaryngology, sevoflurane induction, spontaneous ventilation anaesthesia

## Abstract

Bilateral vocal cord paralysis is a potentially life-threatening condition, and when an agitated patient refuses awake airway management for an emergent procedure, balancing spontaneous ventilation with adequate sedation is essential. This report presents a 31-year-old male with alcohol use disorder who presented with progressive stridor and dyspnea due to bilateral vocal cord paralysis and posterior glottic stenosis. An awake tracheostomy was initially planned but was not feasible due to severe agitation and refusal to cooperate. Instead, the patient underwent inhalational induction with incremental sevoflurane, supplemented by midazolam, dexmedetomidine, and ketamine, while maintaining spontaneous ventilation. Rocuronium was administered only after tracheostomy placement and was reversed with sugammadex. Postoperative bilateral pneumothoraces and pneumomediastinum were managed conservatively. He was ultimately weaned to a tracheostomy collar with preserved voicing. This case suggests that a carefully titrated inhalational induction that preserves spontaneous ventilation may be a feasible, individualized alternative when awake tracheostomy is not possible in agitated patients with critical airway obstruction.

## Introduction

Although rare, bilateral vocal cord paralysis can rapidly progress to an upper airway obstruction requiring an urgent surgical airway [1]. Bilateral vocal cord paralysis refers to neurogenic immobility of the vocal folds, whereas posterior glottic stenosis is a mechanical fixation of the vocal folds caused by scarring at the posterior commissure, most commonly after endotracheal intubation; the two conditions may coexist, and both narrow the glottic airway [2,3]. Symptoms of stridor and dyspnea typically indicate severe narrowing of the glottic opening, which is managed with surgical interventions like an awake tracheostomy or awake tracheal intubation with local anesthesia [4]. Awake tracheal intubation is considered the safest in patients with difficult airways, as it maintains intrinsic airway tone and spontaneous ventilation until the airway is secured. By contrast, induction of general anesthesia in a patient with a critical, fixed glottic obstruction is particularly hazardous: loss of pharyngeal and laryngeal muscle tone, together with apnea, can convert a partial obstruction into a complete one and precipitate a "cannot intubate, cannot oxygenate" situation [5-7]. If an awake approach is unsuccessful, tracheostomy or cricothyrotomy is the next step [4,8]. However, a relative contraindication to an awake airway approach is an uncooperative patient who cannot tolerate the intervention [4]. Agitation, anxiety, or altered mental status introduces barriers to awake approaches. Thus, the anesthesiologist must balance the need to maintain the patient's airway and spontaneous ventilation with the requirement for adequate operating conditions [9]. We describe the perioperative management of a severely agitated patient with bilateral vocal cord paralysis and critical airway narrowing in whom an awake tracheostomy was not feasible.

## Case presentation

A 31-year-old man with alcohol use disorder, acute pancreatitis, and sickle cell trait presented to the emergency department with progressive stridor, dyspnea, and orthopnea. He had a recent hospitalization for delirium tremens, during which he was intubated for agitation. The next day, he self-extubated and complained of dyspnea and voice changes. Subsequently, he developed worsening dyspnea and stridor, with acute deterioration three days before presentation. Nebulized racemic epinephrine for severe respiratory distress was given. The ENT team performed a bedside flexible fiberoptic laryngoscopy, which revealed bilateral paralyzed vocal cords in the paramedian position with a small glottic opening; no formal measurement of the glottic aperture was recorded. The findings were consistent with bilateral vocal cord paralysis with posterior glottic stenosis. Given the urgency of the airway obstruction, no chest imaging was obtained before surgery. The plan was for an awake tracheostomy under local anesthesia; however, he was severely agitated, refused nebulized lidocaine, and was unable to tolerate lying flat, so he remained seated on the operating room table. On arrival to the operating room, his oxygen saturation (SpO2) was 98% on room air, heart rate was 124 bpm, and blood pressure was 179/107 mmHg.

Glycopyrrolate 0.2 mg IV was administered, and a dexmedetomidine infusion was started at 0.4 mcg/kg/h. Midazolam was given in two 1-mg IV doses approximately 10 minutes apart. Because agitation persisted, ketamine 30 mg IV was added to provide dissociative sedation while preserving airway tone and respiratory drive, followed by inhalational induction with sevoflurane by face mask in stepwise increments. Spontaneous ventilation was maintained throughout, with intermittent gentle assisted mask ventilation as needed; peak inspiratory pressures during assisted breaths reached up to 32 cmH2O. SpO2 remained between 97% and 100% until the airway was secured, and end-tidal carbon dioxide peaked at 52 mmHg before tracheostomy placement. Heart rate ranged from 124 to 150 bpm during the procedure. Direct laryngoscopy and bronchoscopy were performed, and a cuffed 6.0 tracheostomy tube was placed. Only after tracheostomy placement was rocuronium 50 mg administered, and controlled ventilation was then initiated. At the end of the procedure, neuromuscular blockade was reversed with sugammadex 400 mg.

The patient was transported to the post-anesthesia care unit on 4 L/min of supplemental oxygen and remained hemodynamically stable (blood pressure 113/76 mmHg, heart rate 88 bpm, SpO2 100%) and mildly anxious. A chest radiograph obtained immediately after tracheostomy placement revealed bilateral pneumothoraces, new compared with the most recent prior radiograph, measuring 17.7 mm on the left and 6.1 mm on the right, with pneumomediastinum, seen as a lucency tracking along the cardiac silhouette (Figure 1). Because no chest imaging had been obtained between presentation and surgery, it could not be determined with certainty whether these findings were present preoperatively. A repeat radiograph obtained later the same day showed interval improvement, with the pneumothoraces decreasing to 13.8 mm on the left and 4 mm on the right (Figure 2). The pneumothoraces and pneumomediastinum were managed conservatively with observation and serial imaging, and pigtail catheter placement was deferred given their decreasing size. Given the persistent pneumomediastinum, an esophagram was obtained on postoperative day 1 to evaluate for esophageal perforation; water-soluble contrast passed through the esophagus without extravasation, filling defect, or evidence of a fistulous tract, excluding esophageal injury (Figure 3).

**Figure 1:**
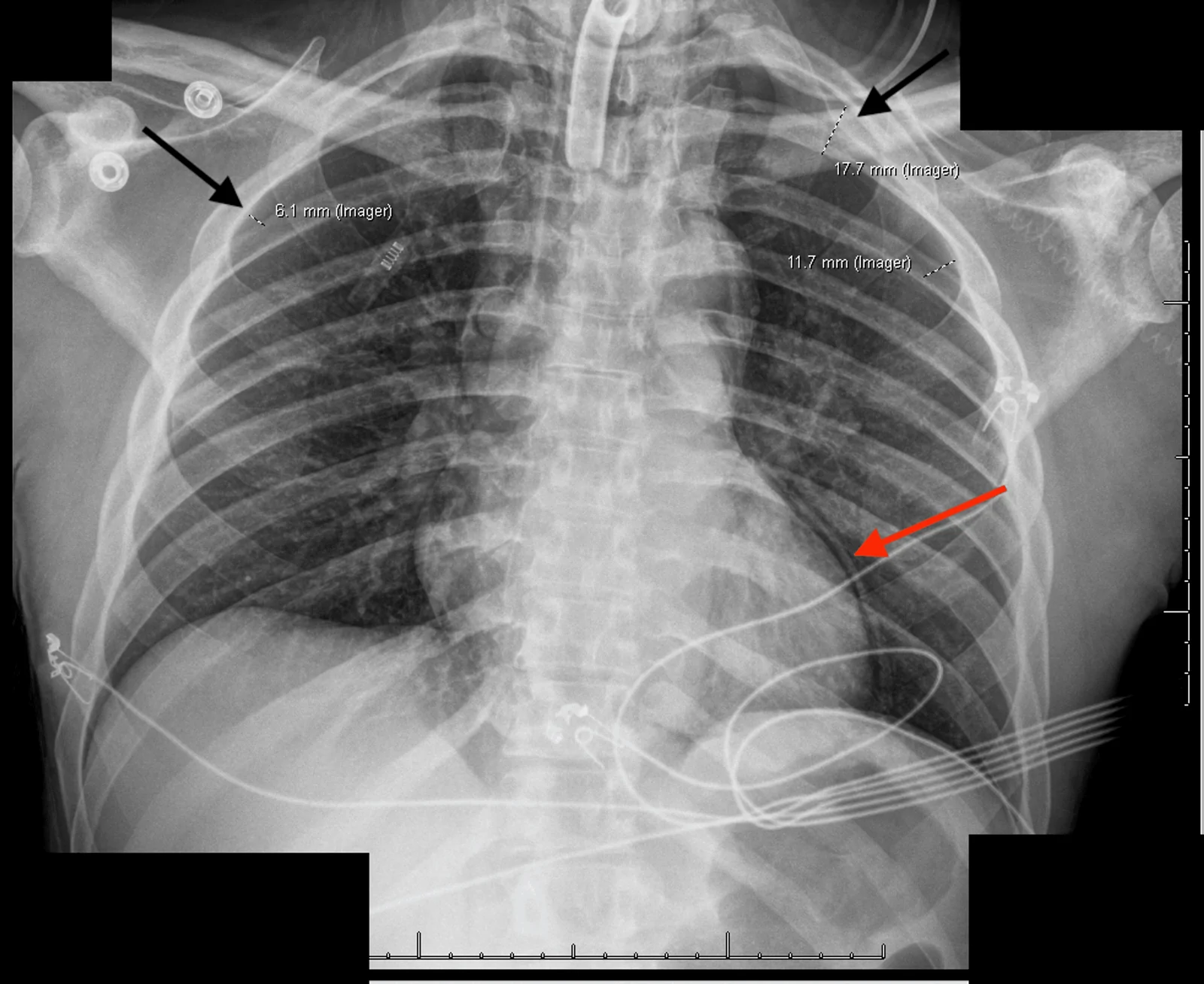
Anteroposterior portable chest radiograph immediately after tracheostomy placement, with new bilateral pneumothoraces (black arrows; 17.7 mm left, 6.1 mm right) with pneumomediastinum (red arrow). Measurements were obtained by the radiologist.

**Figure 2:**
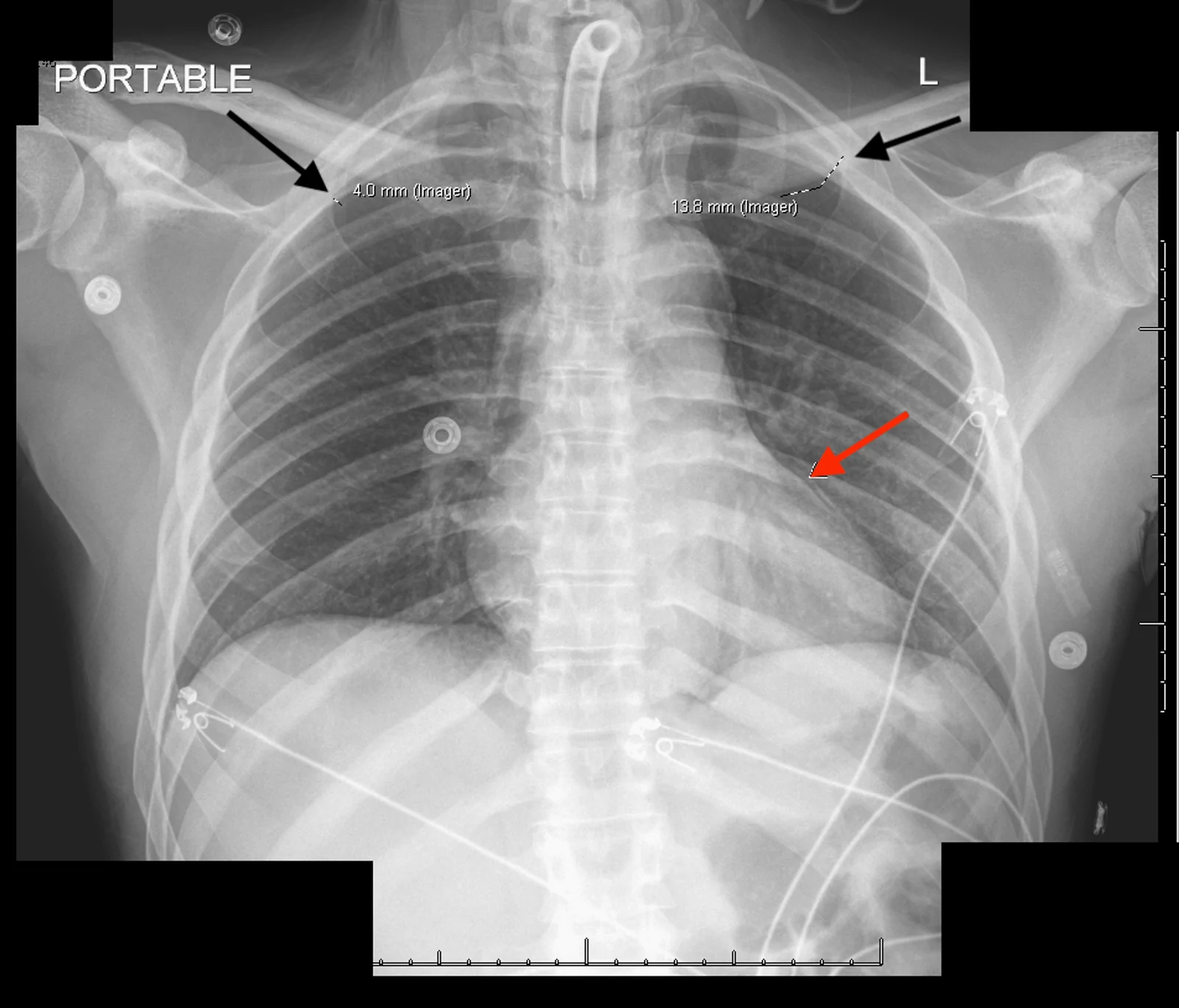
Repeat anteroposterior portable chest radiograph post-tracheostomy placement obtained later the same day, showing interval decrease in the bilateral pneumothoraces (black arrows; 13.8 mm left, 4 mm right), with persistent pneumomediastinum (red arrow). Measurements were obtained by the radiologist.

**Figure 3:**
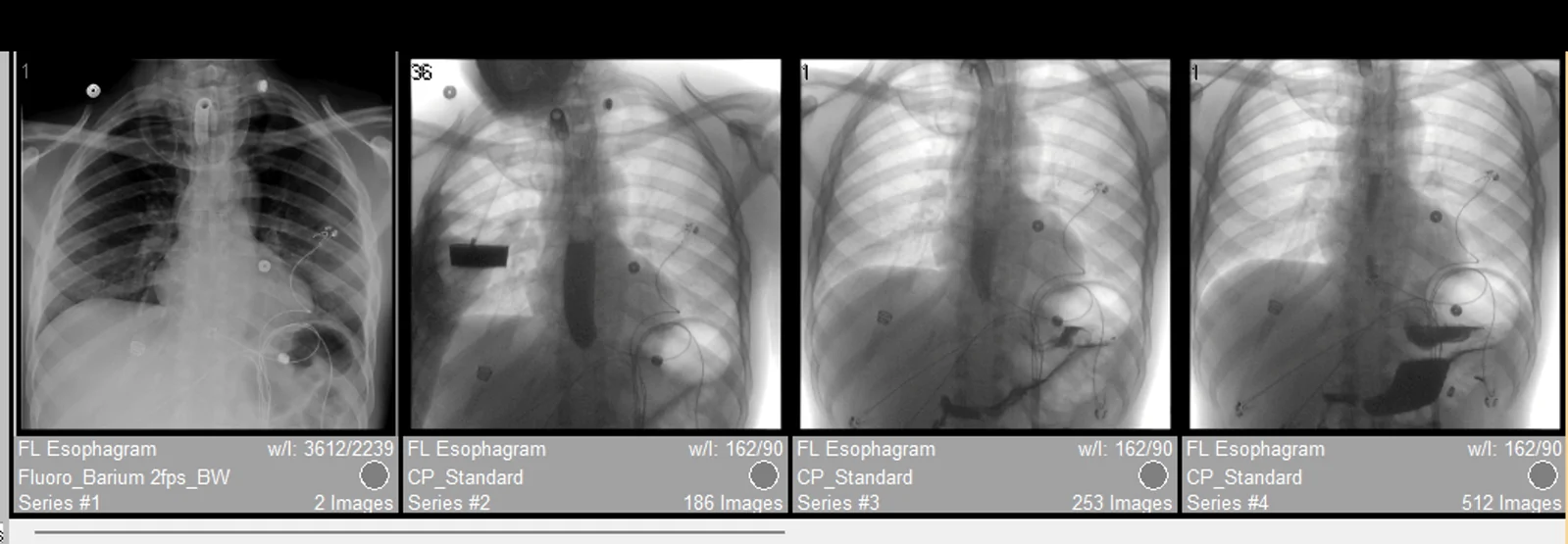
Fluoroscopic esophagram obtained on postoperative day 1 without extravasation, filling defect, or fistulous tract, excluding esophageal perforation.

The hospital course was complicated by alcohol withdrawal with agitation and hypertensive urgency, requiring upgrade to the medical intensive care unit on postoperative day 1, where he was mechanically ventilated via the tracheostomy. Propofol and dexmedetomidine infusions and a chlordiazepoxide taper were used to manage this. On postoperative day 5, dexmedetomidine was discontinued, and the patient was weaned off mechanical ventilatory support to a tracheostomy collar and transferred to the general medical floor. A speech pathology evaluation found clear, strong voicing. He was discharged in stable condition on postoperative day 8.

## Discussion

Awake tracheostomy under local anesthesia is generally not feasible in severely agitated patients who refuse cooperation and topical anesthesia. In this case, the patient's acute agitation, refusal of nebulized lidocaine, and inability to tolerate positioning made the awake approach impossible. We used a multimodal anesthetic strategy to balance the competing demands of maintaining spontaneous ventilation while providing adequate sedation and operating conditions.

Awake tracheostomy or awake tracheal intubation under local anesthesia remains the preferred approach for securing a critically compromised airway, as it preserves spontaneous ventilation and intrinsic airway tone until the airway is definitively secured [4,8]. When general anesthesia is judged potentially unsafe, as with a critically obstructed airway, airway society guidance favors this awake approach over early neuromuscular blockade [5,6]. However, awake tracheal intubation depends on a cooperative patient who can tolerate topicalization and positioning, and inability to cooperate is a recognized relative contraindication [4]. In our patient, agitation from alcohol withdrawal, having recently required intubation for delirium tremens, made the awake approach unworkable, as his psychomotor agitation and inability to cooperate prevented safe topicalization and instrumentation.

Case reports of urgent surgical airway access in similarly uncooperative or non-awake-technique-eligible patients are sparse but instructive. Smith and Fallon reported an uncooperative trauma patient with a difficult airway in whom awake tracheostomy could not be performed, and who instead underwent successful sevoflurane mask induction with preserved spontaneous ventilation to secure the airway [9], demonstrating that carefully titrated volatile induction can serve as a bridge when awake methods are not feasible. In a large single-center series, Fang et al. reported that emergent awake tracheostomy, when feasible, carries an acceptable complication profile, reinforcing that the awake technique remains first-line whenever a patient can tolerate it [10]. Our case extends this literature by combining incremental sevoflurane with several respiratory-sparing adjuncts, reflecting a broader shift toward multimodal induction strategies; McClelland and Hardman argued that inhalational induction remains underused in adults despite its capacity to preserve airway patency throughout induction [11].

The choice of individual agents in this case was rooted in their known respiratory-sparing pharmacology. Ketamine is unique among induction agents in that it activates central respiratory drive and preserves upper airway dilator muscle tone, effectively uncoupling loss of consciousness from the airway collapse typically produced by other induction agents, a mechanism demonstrated by Eikermann et al. in a physiologic model of anesthetic-induced airway dysfunction [12]. Dexmedetomidine similarly provides sedation and anxiolysis without significant respiratory depression; in a randomized trial, Sivakumar et al. found that dexmedetomidine produced intubating conditions comparable to a midazolam-ketamine combination during awake fiberoptic intubation in difficult-airway patients while preserving spontaneous ventilation throughout [13]. Combining these agents with low-dose midazolam likely allowed our patient to tolerate laryngoscopy and bronchoscopy without abolishing his respiratory drive.

Rocuronium was administered only after the tracheostomy tube had been placed to optimize surgical conditions and was reversed with sugammadex at the end of the procedure, allowing rapid restoration of neuromuscular function and return of full spontaneous ventilation. Deferring neuromuscular blockade until the airway was secured was central to the strategy, as paralysis before that point would have removed the patient's only means of ventilation across the critically narrowed glottis. Sugammadex offers a pharmacologic advantage over cholinesterase inhibitors in this context because it directly encapsulates rocuronium rather than depending on residual acetylcholinesterase activity, allowing more rapid and reliable reversal even from deeper blockade [14]. This capability is particularly valuable in patients whose airway management already relies on preserved spontaneous ventilation, as it minimizes the duration of pharmacologic paralysis.

The bilateral pneumothoraces with pneumomediastinum identified postoperatively were likely multifactorial, and their precise mechanism cannot be established from this single case. One plausible contributor is vigorous inspiratory effort against a fixed, critically narrowed glottis, which generates markedly negative intrathoracic pressure, a mechanism implicated in negative-pressure pulmonary edema and barotrauma, as reviewed by Shaikh et al. [15]. However, several alternative or additive mechanisms remain possible. Air may dissect from the tracheostomy site along peritracheal planes into the mediastinum and pleural spaces, as described by Fikkers et al. [16]; direct laryngoscopy and bronchoscopy may have contributed through airway instrumentation; and intermittent assisted mask ventilation during induction, with peak inspiratory pressures up to 32 cmH2O, may have caused positive-pressure barotrauma. In addition, because no chest imaging was obtained before surgery, a pre-existing air leak cannot be fully excluded. An esophagram without perforation excluded an important alternative source, but it does not establish negative-pressure barotrauma as the cause. Regardless of mechanism, clinicians adopting a preserved spontaneous ventilation strategy in similarly obstructed airways should anticipate and monitor for this complication. Emergency front-of-neck access principles, including prompt recognition of ventilatory compromise and a low threshold for chest imaging, remain directly applicable when this complication is suspected [7].

Finally, the underlying etiology of bilateral vocal cord paralysis and posterior glottic stenosis in this patient was presumed related to his recent intubation and self-extubation, consistent with recognized causes of bilateral vocal fold immobility, which include iatrogenic injury from intubation, surgical trauma, and idiopathic or neurologic causes [1,2]. Gardner similarly emphasized that posterior glottic stenosis frequently coexists with, or mimics, bilateral vocal fold paralysis and complicates both diagnosis and airway planning [3]. Recognizing this etiologic overlap is important, as it may inform anticipated difficulty with awake techniques and reinforce the need for a flexible, multimodal backup plan such as the one used here.

## Conclusions

Securing the airway in a patient with critical bilateral vocal cord paralysis and posterior glottic stenosis is inherently high-risk, and this risk is compounded when severe agitation precludes the awake technique that is normally considered safest. This single case demonstrates the feasibility of a carefully titrated, multimodal anesthetic that combined incremental inhalational induction with ketamine, dexmedetomidine, and midazolam, agents chosen for their capacity to preserve respiratory drive and airway tone, and that deferred neuromuscular blockade until the tracheostomy was in place. As a single report, it cannot establish that this approach is broadly effective or safe; rather, it represents an individualized alternative when standard awake airway management is not possible. The postoperative bilateral pneumothoraces with pneumomediastinum, likely multifactorial in mechanism, highlight that preserving spontaneous ventilation does not eliminate risk; clinicians who adopt this strategy should anticipate air-leak complications, maintain a low threshold for postoperative chest imaging, and be prepared to manage them promptly. Close interdisciplinary coordination between anesthesiology, otolaryngology, and critical care, together with individualized titration of sedative and dissociative agents to the patient's hemodynamic and respiratory status, remains central to a successful outcome in this population.
